# A multi-institutional series of a novel, recurrent *TRIM24::MET* fusion-driven infant-type hemispheric glioma reveals significant clinico-pathological heterogeneity

**DOI:** 10.1186/s40478-024-01817-9

**Published:** 2024-06-21

**Authors:** David Gorodezki, Jason Chiang, Angela N. Viaene, Philipp Sievers, Simone Schmid, Ursula Holzer, Frank Paulsen, Martin U. Schuhmann, Olaf Witt, Jens Schittenhelm, Martin Ebinger

**Affiliations:** 1 Department of Hematology and Oncology, University Children’s Hospital Tübingen, Tübingen, Germany; 2 Department of Pathology, St. Jude Children’s Research Hospital, Memphis, TN USA; 3 Department of Pathology and Laboratory Medicine, The Children’s Hospital of Philadelphia, Philadelphia, PA USA; 4 Department of Neuropathology, Institute of Pathology, University Hospital Heidelberg, Heidelberg, Germany; 5 Department of Neuropathology, Charité Universitätsmedizin Berlin, Corporate Member of Freie Universität Berlin and Humboldt-Universität zu Berlin, Berlin, Germany; 6 Department of Radiation Oncology, University Hospital Tübingen, Tübingen, Germany; 7 Section of Pediatric Neurosurgery, Department of Neurosurgery, University Hospital Tübingen, Tübingen, Germany; 8 Hopp Children’s Cancer Center Heidelberg (KiTZ), Heidelberg, Germany; 9 Clinical Cooperation Unit Pediatric Oncology, German Cancer Research Center (DKFZ), Heidelberg, Germany; 10 Department of Pediatric Oncology, Hematology and Immunology, Heidelberg University Hospital, Heidelberg, Germany; 11 National Center for Tumor Diseases (NCT), Heidelberg, Germany; 12 Department of Neuropathology, Institute of Pathology, University Hospital Tübingen, Tübingen, Germany

**Keywords:** Infant-type hemispheric glioma, MET fusion, *TRIM24::MET* fusion

## Abstract

Within the past decade, incremental integration of molecular characteristics into the classification of central nervous system neoplasms increasingly facilitated precise diagnosis and advanced stratification, beyond potentially providing the foundation for advanced targeted therapies. We report a series of three cases of infant-type hemispheric glioma (IHG) involving three infants diagnosed with neuroepithelial tumors of the cerebral hemispheres harboring a novel, recurrent *TRIM24::MET* fusion. Histopathology showed glial tumors with either low-grade or high-grade characteristics, while molecular characterization found an additional homozygous *CDKN2A/B* deletion in two cases. Two patients showed leptomeningeal dissemination, while multiple supra- and infratentorial tumor manifestations were found in one case. Following subtotal resection (two cases) and biopsy (one case), treatment intensity of adjuvant chemotherapy regimens did not reflect in the progression patterns within the reported cases. Two patients showed progression after first-line treatment, of which one patient died not responding to tyrosine kinase inhibitor cabozantinib. As the detection of a recurrent *TRIM24::MET* fusion expands the spectrum of renowned driving fusion genes in IHG, this comparative illustration may indicate a distinct clinico-pathological heterogeneity of tumors bearing this driver alteration. Upfront clinical trials of IHG promoting further characterization and the implementation of individualized therapies involving receptor tyrosine kinase inhibition are required.

## Introduction

Within the past decade, incremental integration of molecular characteristics into the classification of CNS neoplasms increasingly facilitated precise diagnosis and advanced stratification, while moreover fostering a more profound understanding of the distinct biology and oncogenesis of various CNS tumors [1–3]. Comprehensive genetic profiling of pediatric CNS tumors recently facilitated the discovery of a novel type of diffuse pediatric high-grade glial tumor, being recognized as infant-type hemispheric glioma (IHG) in the 5th edition of the WHO Classification of Tumors of the Central Nervous System published in 2021 [1, 4–8]. Occurring in newborns and infants, these glial tumors are characterized by a distinct molecular profile driven by kinase fusion genes involving the *NTRK* family, *ALK, ROS1* or *MET* [4, 6–8]. Notable, these kinase fusion-driven tumors are considered to bear a significantly superior outcome compared to other types of diffuse pediatric-type high-grade glioma, presumably indicating a disparity of histological grading and biological behavior in IHG [4, 6, 9]. Previous series analyzing the genetic landscape of fusion-driven IHG identified *ALK* and the *NTRK* family as the most frequently targeted genes, while recurrent fusions including *ETV6::NTRK3, TPM3::NTRK2, PPP1CB::ALK* and *EML4::ALK* are observed [6]. In contrast, *MET* fusion genes were detected in comparatively rare cases of IHG, showing a prevalence of approximately 6–7% in previous reports [4, 6, 8].

The *MET* proto-oncogene encodes a receptor tyrosine kinase assigned a multifaceted role in tumorigenesis, survival, evasion and dissemination of cancer cells in a diverse spectrum of malignancies, as aberrant signaling due to mutational activation, amplification and overexpression has been described in various carcinomas, sarcomas and melanomas, as well as hematologic and central nervous system malignancies 10–14]. While receptor tyrosine kinase (RTK) signaling pathways including MET are among the most common dysregulated pathways in human gliomas, oncogenic activation of MET due to alterations including *MET* exon 14 skipping or *PTPRZ1::MET* fusion appear significantly enriched in secondary glioblastoma in adult cohorts [15, 16]. MET expression has moreover been shown to correlate with tumor grade in human gliomas and has repeatedly been shown to enhance MAP kinase pathway signaling in glial and glioneuronal tumors [15, 17–19]. Among pediatric CNS neoplasms, *MET* gene fusions have to this day exclusively been reported in the context of infantile high-grade gliomas, while a distinctive enrichment in hemispheric tumors has been observed and recurrent *MET* gene fusions as *PTPRZ1::MET* have been reported [6, 9, 19, 20].

Recently, the detection of the novel *TRIM24::MET* fusion in a particularly aggressive high-grade glial tumor in a neonate has been reported for the first time [21]. In this work, we contribute a multi-institutional series of three further cases of IHG harboring this particular RTK gene fusion, aiming to comparatively illustrate the clinico-pathological characteristics of *TRIM24::MET* fusion-driven IHG and expand the spectrum of renowned recurrent *MET* gene fusions in pediatric gliomas.

## Clinical features

**Fig. 1:**
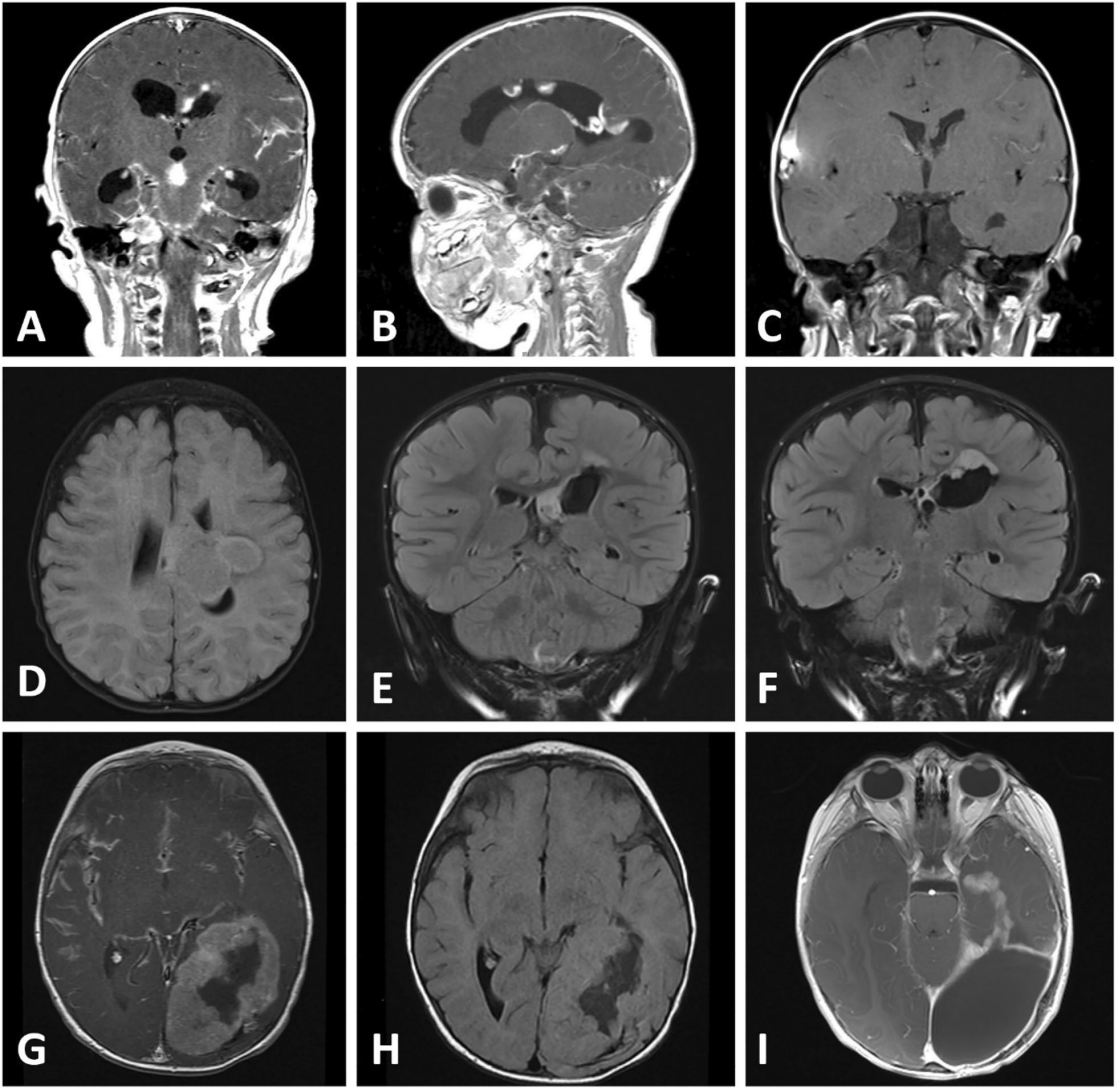
Magnet resonance imaging of the tumors found in the reported cases. **A** Multiple inhomogeneously contrast-enhancing tumors with a maximum expansion of 10 × 20 mm and **B** an infratentorial tumor manifestation in the right cerebellopontine angle with extension in the internal auditory meatus were found in the first case. **C** Follow-up contrast enhanced T1 MRI scan of the first case 3 years after diagnosis showing a stable residual in the right lateral ventricle adjacent to the septum pellucidum without contrast enhancement. **D** T2 flair MRI scan of case 3 at initial diagnosis showing a large heterogeneous mass arising from the deep white matter of the posterior left frontal lobe with extension into the left lateral ventricle. **E** and **F** Coronal T2 flair MRI images of case two attained approximately 2 years after initial diagnosis showing a multinodular recurrence in close proximity to the left lateral ventricle. Contrast enhanced T1 (**G**) and T2 flair (**H**) MRI scans of case 3 at initial diagnosis showing a large tumor involving the left parietal, occipital and temporal lobe with extensive intratumoral hemorrhage. **I** Follow-up MRI scan six months after initial therapy showing a progression of the diffuse contrast enhancing residual tumor

The first case involves a male newborn presenting with desaturations and recurrent neonatal seizures 3 h postnatal. An immediately performed ultrasound and MRI examination on the 7th day of life revealed the suspicion of a grade I intraventricular hemorrhage. Due to the demarcation of multiple hyperechogenic intraventricular lesions with decompensated CSF circulation during follow-up, a repeat MRI scan at 17 weeks confirmed the diagnosis of multiple contrast-enhancing intraventricular tumors with an infratentorial manifestation in the left cerebellopontine angle and diffuse leptomeningeal enhancement (Fig. 1A, B). The patient was subsequently referred to a tertiary care hospital for endoscopic tumor biopsy and placement of a ventriculoperitoneal shunt. At the time of diagnosis, the patient showed no neurological symptoms and remained seizure-free under anticonvulsant treatment with phenobarbital and levetiracetam. Due to disseminated disease and young age, a low-toxicity profile chemotherapy regimen with weekly vinblastine applications over the course of 18 months was conducted, showing an immediate and sustained response with minor, stable intraventricular tumor residuals without contrast enhancement in successive follow-up MRI examinations 3 years after initial diagnosis (Fig. 1C).

The second tumor was diagnosed in a 5-month-old female presenting with seizures, while a MRI scan revealed a large heterogeneous mass with internal calcifications arising from the deep white matter of the posterior left frontal lobe, with extension into the left lateral ventricle, showing restricted diffusion and contrast enhancement (Fig. 1D). No evidence of leptomeningeal metastases was present. The patient underwent subtotal resection at the age of 5 months, and the tumor was classified as a high-grade glioma. Due to occurrence of two small, residual nodules at the primary tumor site during follow-up, chemotherapy according to CCG-9921 protocol including an induction regimen consisting of vincristine, carboplatin, ifosfamide and etoposide was conducted, followed by a maintenance chemotherapy regimen consisting of vincristine, etoposide, carboplatin and cyclophosphamide [22]. Due to tumor progression at the age of 2 years (Fig. 1E–F), the patient underwent secondary resection and was subsequently treated with an adjuvant therapy of 8 courses of CCNU/temozolomide. There is no evidence of tumor recurrence 5 years after second resection.

The third case involves a male patient presenting with lethargy, vomiting and a focal seizure at the age of 7 months. An initially performed MRI scan revealed a large complex hemispheric T1 isointense, T2 hyperintense and contrast-enhancing tumor involving the left parietal, occipital and temporal lobe with a maximum expansion of 7.3 × 4.6 × 5.9 cm (Fig. 1G, H). The tumor showed circumscribed margins, restricted diffusion and extensive intratumoral hemorrhage without calcifications. There was evidence of leptomeningeal dissemination. The patient was subsequently transferred to a tertiary referral center for further treatment. After an initial biopsy, near-total resection was performed, followed by an adjuvant chemotherapy regimen in accordance with the study protocol of the SJYC07 trial, including an induction with high-dose methotrexate, vincristine, cisplatin and cyclophosphamide, followed by a consolidation with cyclophosphamide [23]. Due to recurrent infections including a fungal infection, consolidation therapy was discontinued after two cycles. After an observation period of approximately 6 months, a secondary incomplete resection due to radiological progression was performed, followed by a targeted therapy with cabozantinib. After three months of stable disease, the patient died approximately 17 months after initial diagnosis due to recurrent tumor progression (Fig. 1I).

## Histopathology

**Fig. 2:**
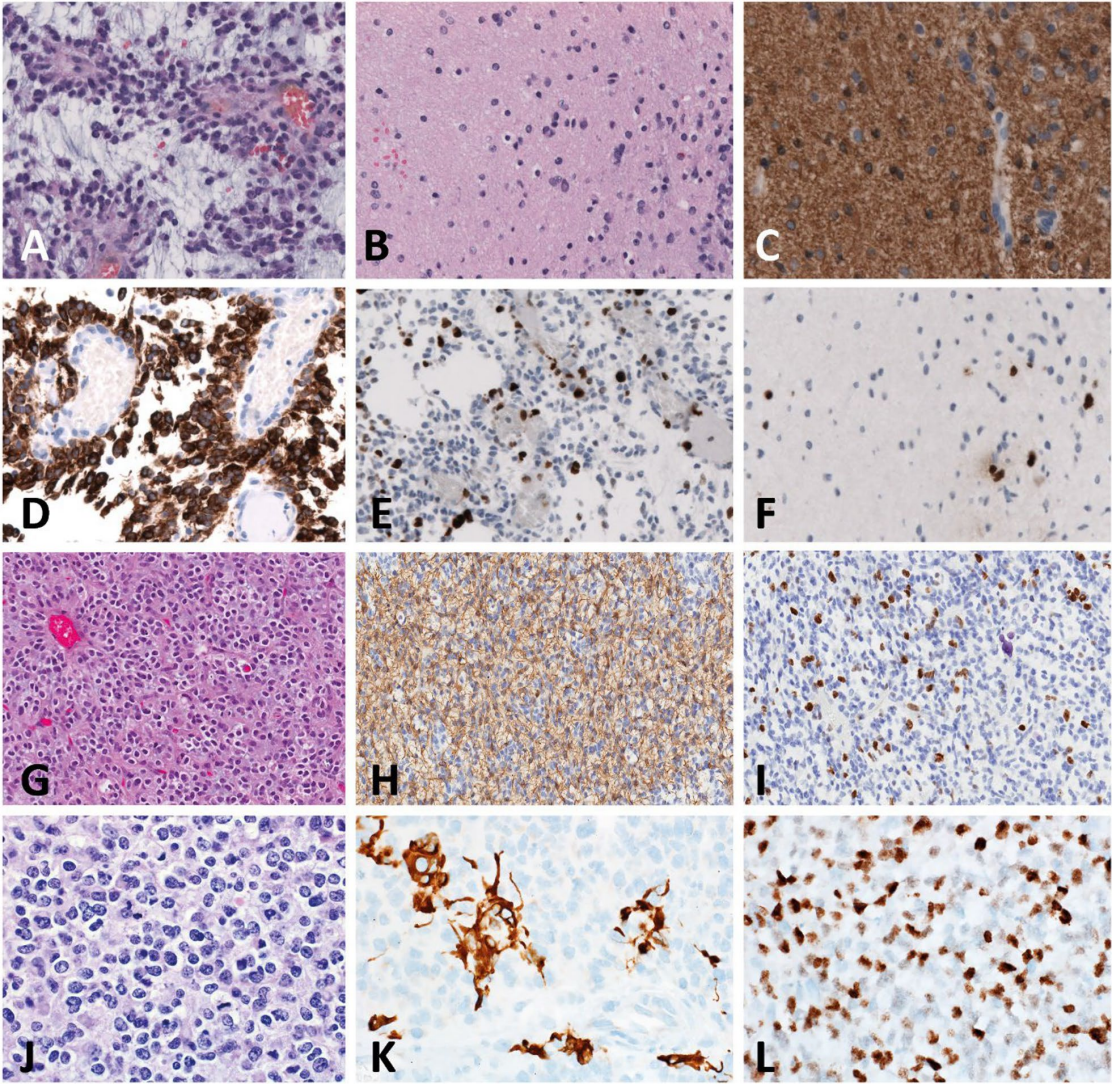
Histologic appearance of the tumors found in the reported cases. **A** Hematoxylin and eosin (H&E) stain of papillary regions of the tumor in case 1 showed diffuse interstitial myxoid debris.  **B** H&E stain of diffuse regions of the tumor found in case 1 showing a moderate cellular density and mild nuclear pleomorphism. No microvascular proliferations or (palisading) necrosis were present. GFAP stain of diffuse tumor regions **C** and papillary tumor regions **D** of the tumor in case 1 showed regional positivity. MIB-1 immunostaining of the tumor in case 1 showed positivity in up to 10% of cells in papillary tumor regions **E** and 3–5% in diffuse tumor regions **F**. **G** H&E stain of the tumor found in case 2 showing a neuroepithelial neoplasm with sheet-like growth pattern within a fibrillary background with high cellular density, scattered mitoses and small calcifications. **H** GFAP immunostaining of the tumor found in case 2 showed a positivity for a subset of cells. **I** The Ki-67 proliferation index in case 2 was approximately 10%. **J** H&E stain of the tumor found in case 3 showing a neuroepithelial mass of homogeneously composed small cells with sheet-like growth pattern and high cell density. **K** GFAP immunostaining of the tumor found in case 3 showed a regional positivity for a subset of cells. **L** The Ki-67 proliferation index in case 3 exceeded 25%

Pathologic review following endoscopic biopsy of the tumor found in the first case revealed a papillary neuroepithelial tumor with mainly ovoid cells, moderate cellular density, angiocentric growth patterns and prominent interstitial myxoid debris. Neither microvascular proliferations nor palisading necrosis were apparent, only very few mitotic figures could be detected. Immunohistochemistry showed regional positivity for GFAP and diffuse expression of MAP2, while no specific immunoreactivity for IDH1(R132H) or H3(K27M) could be detected. Nuclear ATRX expression was retained. The MIB-1 (Ki-67) labeling index was approximately 5–10%. The tumor was therefore initially classified as a low-grade glial tumor (Fig. 2A–F).

Histopathologic review of the second tumor showed a well-circumscribed appearance. No areas of infiltration were observed, while calcifications and scattered mitoses were present throughout the tumor. The tumor was composed of small to medium-sized glial cells with sheet-like growth patterns within a myxoid background. Tumor cells were positive for Olig2 and GFAP, while immunohistochemical staining for neuronal markers was negative. MIB-1 (Ki-67) proliferation index was up to 10%; the tumor was classified as a high-grade glioma. The recurrent tumor showed a significantly lower cell density and a lower MIB-1 (Ki-67) proliferation index, accounting for approximately 1% (Fig. 2G–I).

Pathological review after near-total resection of the tumor found in case three showed a homogeneous composition of small glial cells with high cell density and sheet-like growth pattern. Tumor cells showed regional immunohistochemical positivity for glial markers as Olig2 and GFAP, MIB-1 (Ki-67) proliferation index exceeded 25%. Based on the histopathological appearance, the tumor was initially classified as a high-grade neuroepithelial tumor, while no further subclassification could be achieved (Fig. 2J–L).

## Molecular characterization

**Fig. 3:**
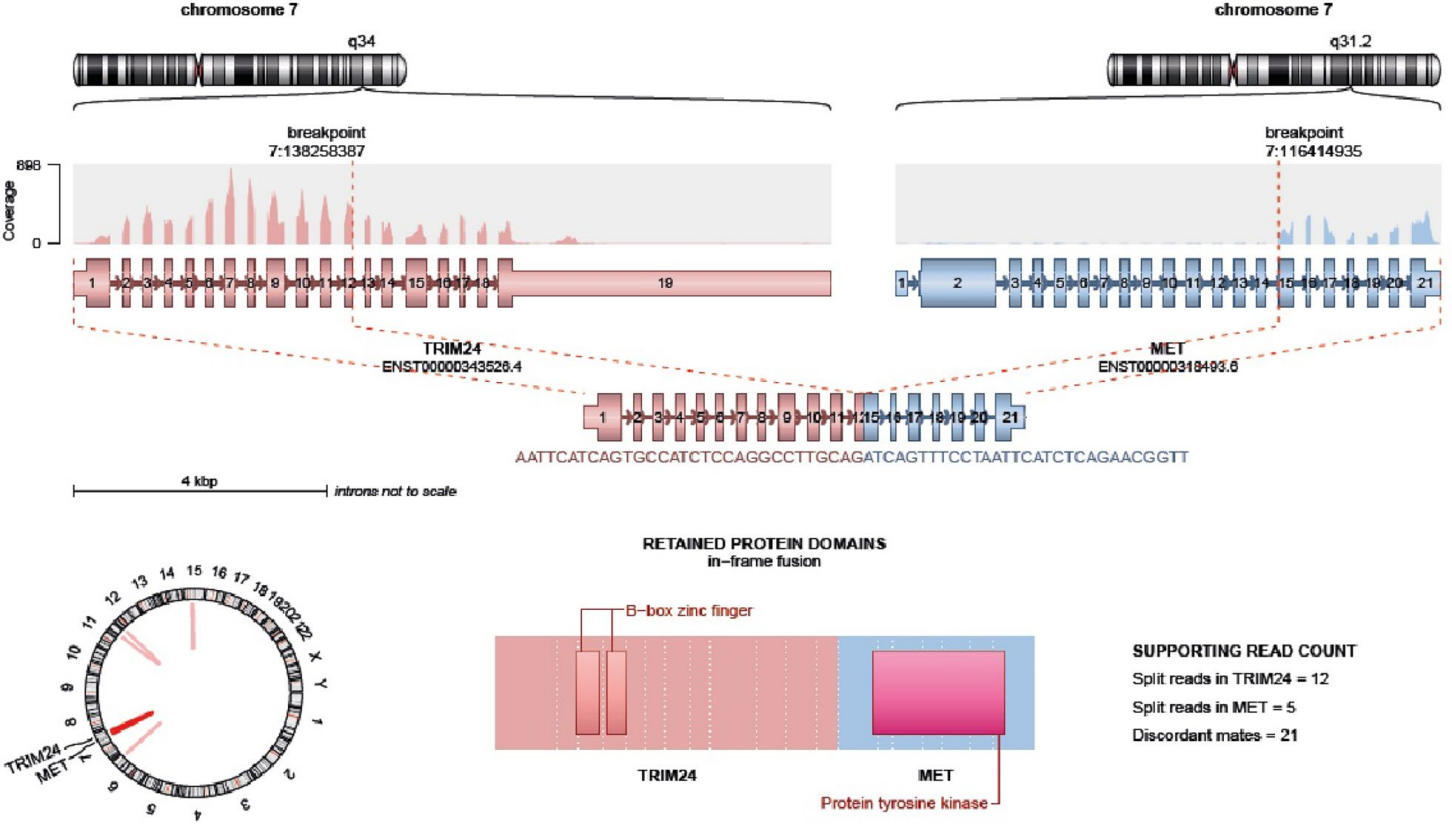
Illustration of the recurrent *TRIM24::MET* gene fusion between exon 12 of *TRIM24* and exon 15 of *MET* on chromosome 7 involving the MET tyrosine kinase domain found in the tumors of all three reported cases involving similar breakpoint regions

DNA methylation array analysis of the first tumor was performed following DNA extraction from formalin-fixed, paraffin-embedded tumor tissue using the Infinium MethylationEPIC (850 k) Array (Illumina) and compared to DNA methylation data of a reference cohort comprising over 2.800 neuropathological tumors of 82 entities (www.molecularneuropathology.org; classifier version v11b4). Although no significant conformity with any established DNA methylation class could be observed, the highest classifier score (0.7) was reached for infant-type hemispheric glioma. For the purpose of this study, we repeated methylation array profiling using the latest MNP brain tumor classifier version (v12.8), which again showed a subthreshold score (0.54) with the highest conformity to IHG. Although the amount of tissue available appeared marginally sufficient for the analysis, DNA concentration after extraction appeared sufficient for the EPIC analysis (estimated tumor cells: 70%, DNA concentration: 30 ng/µl, EPIC yield 500 ng). A copy number profile showed an indication for a partial loss of distal chromosome 9p and a deletion of *CDKN2A/B*. Next generation sequencing (NGS) of tumor DNA and RNA was performed as previously described in collaboration with a reference laboratory of the LOGGIC Core BioClinical Data Bank, and results were obtained from kindly provided reference pathology reports [24]. NGS of DNA was performed using the NPHD gene panel (version 2019A) including 160 significant genes related to CNS tumors, as previously reported [25]. No pathogenic or likely pathogenic alterations could be found. Two variants of uncertain significance were detected (*ATM* c.G5262T; *KMT2C* c.T2959C). Subsequent NGS of tumor RNA identified a *TRIM24::MET* fusion between exon 12 of *TRIM24* and exon 15 of *MET* on chromosome 7 involving the *MET* tyrosine kinase domain (Fig. 3). In alignment with the DNA methylation data, the diagnosis of IHG was assigned.

In the second case, RNA-based NGS performed on the recurrent tumor revealed a *TRIM24::MET* fusion between exon 12 of *TRIM24* (NM_015905.2) and exon 15 of *MET* (NM_000245.3) and a gain of chromosome 7 without any evidence of further genetic alterations, especially no signs of *CDKN2A/B* deletion. The findings were consistent with an integrated diagnosis of IHG. At the time of diagnosis methylation array profiling was performed in this case, and additional studies cannot be performed due to lacking material.

**Table 1:** Comparison of imaging features, histopathologic appearance, molecular data, therapy regimens and clinical course of the three presented cases of IHG harboring a *TRIM24::MET* fusion

	Case number		
	1	2	3
Imaging features	Disseminated tumors with CE in lateral ventricles and cerebellopontine angle, diffuse LME	Diffusion restricted tumor with CE in the left frontal lobe and lateral ventricle, internal calcifications, no LME	Heterogeneous well-circumscribed tumor with CE in left parietal, occipital and temporal lobe, diffuse LME
Histopathology	Papillary neuroepithelial tumor, moderate cell density. MIB1-LI 5%. GFAP and MAP2 positive, classified as LGG	Well circumscribed glial tumor, calcifications, GFAP and Olig2 positive, MIB1-LI 10%, classified as HGG	Homogeneous glial tumor, high cell density, GFAP and Olig2 positive, MIB-1 LI > 25%, classified as LGG
DNA methylation profile (v18.2)	No significant conformity, highest classifier score: IHG (0.54)	n.a	No significant conformity, cluster in proximity to IHG on t-SNE brain tumor world map
Molecular profile	*TRIM24::MET* fusion, homozygous *CDK2NA* deletion	*TRIM24::MET* fusion	*TRIM24::MET* fusion, homozygous *CDK2NA* deletion
Extent of surgery	Biopsy only	Incomplete resection (IR), secondary IR after progression	Biopsy followed by IR, secondary IR after progression
Adjuvant therapy	VBL weekly for 18 months	Induction: VCR, CB, IFO and VP-16. MT: VCR, VP-16, CB and CPM (CCG-9921) After progression: CCNU/TMZ	Induction: HD-MTX, VCR, CP and CPM. MT: CPM (SJYC07) After progression: carbozantinib
Outcome	Treatment response, minor tumor residuals, Progression-free 3 years after diagnosis	PD 2 years after first-line treatment, progression-free 5 years after 2nd resection	PD 6 months after initial therapy, PD 3 months after 2nd treatment, died 17 months after diagnosis

*CE* contrast enhancement, *LME* leptomeningeal enhancement, *MT* maintenance therapy, *PD* progressive diasease, *VBL* vinblastine, *VCR* vincristine, *CB* carboplatin, *IFO* ifosfamide, *VP-16* etoposide, *CPM* cyclophosphamide, *HD-MTX* high-dose methotrexate

In case three, DNA methylation array profiling showed no significant conformity with any established DNA methylation class. For the purpose of this study, we repeated methylation array profiling using the latest MNP brain tumor classifier version (v12.8), which again showed no significant conformity to any established DNA methylation class, while a proximity to Paediatric-type diffuse high-grade gliomas, was found (score: 0.61). NGS, performed after second surgery, revealed a *TRIM24::MET* fusion between exon 12 of *TRIM24* and exon 15 of *MET* involving the *MET* tyrosine kinase domain, leading to the integrated diagnosis of IHG. In addition, a homozygous *CDKN2A* deletion was detected. Imaging features, histopathologic and molecular characteristics, treatment patterns and outcome of the reported cases is summarized in Table 1.

## Discussion

Within the past decade, the differentiation of a novel infant-type hemispheric glioma emerged from comprehensive molecular characterization of high-grade gliomas in very young children, representing neonatal and infant tumors characterized by a distinct molecular profile driven by kinase fusion genes involving the *NTRK* family, *ALK*, *ROS1* or *MET* [4, 6–8]. To this day, limited experience and few published series translate to uncertainty and heterogeneity concerning the clinical management of these tumors, while comprehensive stratification and standardized treatment recommendations are missing.

The detection of *MET* fusion genes were reported in comparatively rare cases of IHG [4, 6, 8]. While the discovery of a novel *TRIM24::MET* fusion in a particularly aggressive high-grade glial tumor in a neonate has recently been reported for the first time, the present work contributes three further cases of IHG harboring this particular RTK gene fusion characterized by a similar region of breakpoints and conservation of the tyrosine kinase domain [21]. Comparative illustration of the reported cases indicates notable differences in tumor dissemination, histopathologic appearance and tumor progression patterns, which, however, may be confounded by several variables including significant differences in the extent of surgical resection and the intensity of adjuvant chemotherapy regimens.

Based on histological characteristics, the present cases were classified both as low-grade (case one) and high-grade (cases two and three) neuroepithelial tumors. This may reflect the variety of histological appearance of IHG, consistent with previously published series reporting a primary diagnosis of high-grade glioma in approximately 80% of IHG cases, whereby a variety of other diagnoses were included in the original pathology reports [5, 6]. Notably, despite its designation as a subform of pediatric-type diffuse high-grade gliomas in the fifth CNS WHO classification, a formal WHO grade has not yet been assigned to IHG, while based on current experience, the absence of high-grade features appears compatible with this diagnosis [26]. As *MET* fusions previously have exclusively been described in high-grade gliomas in the context of pediatric CNS tumors, the detection as a driver alteration in a glioneuronal tumor bearing low-grade histological characteristics and a benign clinical course (case one) may expand its spectrum of infant glioma phenotypes. Remarkably, a previous series reported a decrease of histological grade from consecutive biopsies in several cases of IHG, suggesting that these tumors may either comprise a low-/high-grade continuum or bear the potential to maturate over time, as previously demonstrated in the context of oncogene-induced senescence in MAPK-driven pediatric gliomas [4, 27, 28]. This may be supported by the previous observation of a strong MAPK activation due to MET overexpression in a MET fusion-positive pediatric glioma in vitro model [19].

In two cases (cases one and three), tumors harbored a homozygous *CDKN2A/B* deletion, consistent with a previously published series and observations in xenograft models, suggesting a dependency of *MET* fusion-induced tumorigenesis on additional cell cycle regulation impairing genetic lesions in pediatric glioma [19].

The tendency towards diverging adjuvant treatment regimens may reflect both the diagnostic uncertainty as well as missing experience and specific treatment recommendations in the context of IHG. In line with histological presentation, a low-toxicity profile vinblastine-based chemotherapy regimen was applied in the first case, while intensified alkylator-based chemotherapy regimens according to CCG-9921 (second case) and SJYC07 (third case) study protocols were applied as first-line treatment in cases two and three. Both protocols were previously implemented to withhold radiation therapy before tumor progression in young children [22, 23]. The intensity of the applied first-line chemotherapy regimens, however, is not reflected by the outcome of the reported cases, and universal treatment recommendations cannot be derived due the limited number of reported cases and an inconclusive heterogeneity of applied treatment protocols and subsequent progression patterns. Beyond, the possible confounding effect of several variables on outcome comparison including diverging treatment patterns among the reported cases should be considered.

Despite limited experience and few published series, previous data suggest a superior outcome of IHG compared to distinct high-grade glioma types of pediatric cohorts [4]. Whether the type of RTK gene fusion has prognostic implications and distinct fusion genes may yield certain outcomes in IHG has yet to be addressed in prospective clinical studies. The present comparative illustration of *TRIM24::MET* fusion positive IHG, however, may indicate a notable heterogeneity of clinical outcomes including both the observation of a benign clinical course (case one) and a case of severe therapy resistance and lethal outcome (case three).

The emerging role of a comprehensive integration of DNA methylation array profiling in a multi-layered diagnostic approach was shown to significantly increase diagnostic accuracy, potentially improving survival in a substantial proportion of pediatric CNS tumor patients [29]. Remarkably, discrepant results by neuropathological WHO-based and DNA methylation-based classification appear significantly enriched in histologically high-grade gliomas, while a substantial fraction of tumors could not be confidently assigned to a DNA methylation class in a previously published comprehensive analysis, similar to the findings in the reported cases [29]. It has been reported, that many cases of IHG did not unequivocally classify as either IHG in methylation profiling in a previous series, as found in our cases [6]. While the current reference data set of rather low number of IHG samples has few cases of MET fusion, it does not include cases with this novel *TRIM24::MET* fusion, suggesting that the methylation spectrum of such samples might be larger than anticipated from the initial cohort. In this setting, as illustrated in these cases, the implementation of high-throughput sequencing technologies like RNA-based NGS panels or whole transcriptome sequencing for the identification of potential hallmark molecular driver alterations for precise classification can be of paramount importance [26].

The detection of RTK fusion genes as hallmark molecular driver alterations not only fosters precise classification, but moreover bears the potential for targeted therapies involving RTK inhibition in IHG. Recently, multiple case studies reported significant responses in individual treatment attempts with lorlatinib, entrectinib and larotrectinib in *ALK* and *ROS1* fusion positive IHG, potentially broadening the horizons of curative treatment options within a demographic peculiarly susceptible to severe sequela following conventional salvage treatment including extensive surgery or radiotherapy [9, 30–36]. The suppression of tumor growth by MET inhibition in xenograft models of IHG has previously been demonstrated, and a substantial temporary treatment response in a patient harboring a MET fusion positive pediatric high-grade glioma treated with small molecule MET inhibitor crizotinib has been reported, whereby the appearance of further treatment-resistant lesions has been observed [19]. Despite promising observations, long-term follow-up experience and systematic studies are still missing, while therapy-induced resistance due to molecular evolution is well-described and represents a major challenge in therapeutic approaches involving RTK inhibition, presumably calling for the implementation of combination therapies in upfront clinical trials to achieve durable treatment responses [19, 31].

## Conclusions

As the detection of a recurrent *TRIM24::MET* fusion expands the spectrum of known driving fusion genes in infant-type hemispheric glioma, this comparative illustration may indicate a notable clinico-pathological heterogeneity of tumors bearing this particular molecular driver alteration. Upfront clinical trials of IHG promoting further characterization and the implementation of individualized therapies involving RTK inhibition are required.

## Data Availability

Detailed clinical data, MRI images and original molecular data are available from the corresponding author on reasonable request.

## Abbreviations

CNS: Central nervous system
IHG: Infant-type hemispheric glioma
RTK: Receptor tyrosine kinase
H&E: Hematoxylin and eosin
NGS: Next generation sequencing
IR: Incomplete resection
VBL: Vinblastine
VCR: Vincristine
CB: Carboplatin
IFO: Ifosfamide
VP-16: Etoposide
CPM: Cyclophosphamide
HD-MTX: High-dose methotrexate
